# Test Performance of Cervical Cytology Among Adults With vs Without Human Papillomavirus Vaccination

**DOI:** 10.1001/jamanetworkopen.2022.14020

**Published:** 2022-05-25

**Authors:** Deanna Teoh, Gwiwon Nam, Danielle A. Aase, Ruby Russell, Genevieve B. Melton, Shalini Kulasingam, Rachel I. Vogel

**Affiliations:** 1 Department of Obstetrics, Gynecology & Women’s Health, University of Minnesota, Minneapolis; 2 now with College of Journalism and Communications, University of Florida, Gainesville; 3 now with Department of Obstetrics & Gynecology, Mayo Clinic, Rochester, Minnesota; 4 Institute for Health Informatics, University of Minnesota, Minneapolis; 5 Department of Surgery, University of Minnesota, Minneapolis; 6 Center for Learning Health System Sciences, University of Minnesota, Minneapolis; 7 School of Public Health, Division of Epidemiology and Community Health, University of Minnesota, Minneapolis

## Abstract

**Importance:**

Current US cervical cancer screening guidelines do not differ by human papillomavirus (HPV) vaccination status. However, as the positive predictive value (PPV) of a screening test decreases, the risk of a false-positive result increases.

**Objective:**

To evaluate whether HPV vaccination is associated with decreased PPV for abnormal cervical cytology.

**Design, Setting, and Participants:**

This retrospective cohort study conducted via electronic medical record review included eligible patients aged 21 to 35 years who had at least 1 cervical cytology result within a single health system between January 2015 and December 2018. The health system comprises a partnership between an academic health center and a private not-for-profit health center. Patients with abnormal screening cytology and no diagnostic test results were omitted from analysis. Data were analyzed from December 2019 to November 2021.

**Exposures:**

HPV vaccination, defined as receiving at least 1 dose of HPV vaccine. Subgroup analyses were performed for those completing all vaccination doses per Advisory Committee on Immunization Practices guidelines and by age at vaccination initiation, dichotomized as younger than 21 years vs 21 years or older.

**Main Outcomes and Measures:**

PPV of abnormal cervical cytology for risk of cervical intraepithelial neoplasia (CIN) 2 or more severe diagnosis.

**Results:**

A total of 46 988 patients (mean [SD] age, 28.7 [4.5] years; 3058 [6.5%] Asian; 4159 [8.9%] Black or African American; 35 446 [75.4%] White) were included; 15 494 (33.0%) were at least partially vaccinated, and 4289 (9.1%) had abnormal cytology results during the study period. Among the individuals with abnormal cytology, the PPV for CIN 2 or more severe diagnosis was lower among vaccinated individuals (17.4%; 95% CI, 16.4%-18.4%) than unvaccinated individuals (21.3%; 95% CI, 20.4%-22.3%). Among vaccinated individuals, PPV was significantly lower among those completing vaccination (15.9%; 95% CI, 14.9%-17.0%) than those with incomplete vaccination (22.4%; 95% CI, 20.0%-25.0%), especially among those initiating vaccination when younger than 21 years (11.9%; 95% CI, 10.9%-12.9%) vs those initiating at age 21 years or older (30.7%; 95% CI, 27.3%-34.4%).

**Conclusions and Relevance:**

Among a population with relatively low HPV vaccine coverage, the PPV of cervical cytology for CIN 2 or more severe diagnosis was significantly lower among vaccinated individuals. PPV will likely further decrease in the future as a population with higher HPV vaccination coverage ages into screening. Confirmation of these results will call for changes in screening strategies, particularly for completely vaccinated individuals who initiated vaccination when younger than 21 years.

## Key Points

**Question:**

Is the positive predictive value of abnormal cervical cytology lower among individuals who have been vaccinated against human papillomavirus?

**Findings:**

In a cohort study of 46 988 patients aged 21 to 35 years participating in cervical cancer screening, the positive predictive value of cervical cytology for cervical intraepithelial neoplasia 2 and more severe diagnoses was significantly lower among vaccinated individuals (17.4%) than unvaccinated individuals (21.3%).

**Meaning:**

The observed increased risk of a false-positive cervical cytology result for individuals vaccinated against HPV may warrant screening guidelines that stratify by vaccination status to minimize the risks of overscreening and overtreatment among individuals with low risk for cervical cancer.

## Teaser

This cohort study evaluates whether human papillomavirus (HPV) vaccination is associated with decreased positive predictive value for abnormal cervical cytology.

## Introduction

Vaccination against human papillomavirus (HPV) decreases infection risk with vaccine-type HPV up to 90%, with absolute prevalence of only 1.3% among vaccinated individuals.^1,2^ As expected, prevalence of high-grade cervical dysplasia and cancer has also decreased among vaccinated persons.^3,4,5^ However, protection varies by vaccination coverage within a population, primarily because of differences in cross-protection against non–vaccine type HPV infection. A meta-analysis showed the relative risk of infection with nonquadrivalent HPV vaccine types was 0.73 (95% CI, 0.60-0.89) in populations with vaccination coverage of greater than 50%, and 1.09 (95% CI, 0.67-2.01) with vaccination coverage of less than 50%.^6^ Furthermore, observational studies have shown the risk of cervical intraepithelial neoplasia (CIN) 2 or more severe diagnosis after a single dose of HPV vaccine to be similar to the risk among unvaccinated individuals,^4,7^ although early results from a prospective longitudinal study in India show similar protection for 1 to 3 doses among individuals vaccinated up to 18 years of age,^8^ and studies to determine whether there is a group for whom a single dose of HPV vaccine is adequate are ongoing. Vaccine protection against CIN 2 or more severe diagnosis is also greater for individuals initiating the vaccine series by age 17 years (CIN risk reduction, approximately 40%, cancer risk reduction, approximately 88%) compared with those initiating vaccination at age 20 to 21 years or older (CIN risk reduction, 6%; cancer risk reduction, 42%).^4,5,9^

As prevalence of CIN 2 and more severe diagnoses decrease, the positive predictive value (PPV) of cervical cancer screening tests will decrease.^10,11^ Current cervical cancer screening recommendations do not differentiate by vaccination status. Modeling for screening of vaccinated individuals predicts initiating screening at age 25 to 30 years with an interval of 10 years to be the best strategy.^12^ However, this model assumed vaccination initiation at 12 years of age and prior to HPV exposure as well as completion of the vaccine series with 100% efficacy against vaccine-type HPV infection. Unfortunately, in the United States, only 61.4% of female individuals are currently up-to-date on HPV vaccination by age 17 years.^13^ Thus, additional estimates using clinical data are needed to determine the optimal screening strategy for vaccinated individuals.

The primary objective of this study was to compare the PPV of cervical cytology to diagnose CIN 2 or more severe diagnosis by HPV vaccination status. The secondary objectives were to determine variations in PPV by age at vaccination initiation and number of doses received.

## Methods

### Study Design and Population

This retrospective cohort study was approved by the University of Minnesota institutional review board. Informed consent was waived for this minimal risk study. The study manuscript follows the Strengthening the Reporting of Observational Studies in Epidemiology (STROBE) reporting guidelines for cohort studies. Study participants were individuals aged 21 to 35 years who had undergone cervical cancer screening between January 1, 2015, and December 31, 2018, within the MHealth Fairview System, an academic and not-for-profit health system partnership. The age limits were chosen to exclude persons younger than 21 years who were not yet eligible for cervical cancer screening^14,15^ and to include individuals vaccinated at the upper age limit of 26 years in 2006, when the first HPV vaccines were approved for use in the United States.^16^

The Clinical Data Repository comprising electronic health record (EHR) data of 2.5 million patients was queried to identify eligible participants. The EHR integrates vaccination information from the state vaccination registry. An automated query was first performed using laboratory, *Common Procedural Terminology*, and *International Statistical Classification of Diseases and Related Health Problems, Tenth Revision *codes. The data were preprocessed to identify discrepant or missing data. The natural language processing (NLP) tool NLP-PIER was then applied to abstract unstructured text from pathology notes and health care professional progress notes.^17,18^ Data cleaning was conducted via manual medical record review on an as-needed basis. Discrepancies in data coding were reviewed by 3 investigators (D.A.A. or an independent reviewer, R.R., and D.T.) and resolved by consensus.

### Data Collection and Study Measures

The following data, selected based on associations with cervical cancer screening adherence and cervical cancer risk, were abstracted from the electronic health record: (1) demographic characteristics, including birthdate; race (Asian, Black or African American, White, and other [American Indian or Alaska Native, Native Hawaiian or other Pacific Islander, other race not included in the given categories, and >1 race]); and ethnicity (Hispanic or Latina and not Hispanic or Latina); (2) smoking status (never, former, or current smoker); (3) HPV vaccination status (≥1 dose or none), number of vaccine doses and dates of administration, type of HPV vaccine (HPV-2, HPV-4, or HPV-9); (4) cervical cytology results; and (5) diagnostic test results, including colposcopic findings and biopsy pathology results.

Age at HPV vaccination initiation was calculated. For the primary analysis we defined vaccinated as receiving at least 1 dose of the HPV vaccine, per the definition used most commonly in the literature. Vaccinated was further categorized as completely vaccinated if vaccination was initiated at age 14 years or younger and at least 2 doses were administered, or if vaccination was initiated at age 15 years or older and 3 doses were administered, as recommended by the Advisory Committee on Immunization Practices (ACIP).^19^

Diagnostic test results were categorized as normal if colposcopic impression was normal and no biopsies were taken or if biopsy results showed no dysplastic changes. Low-grade histology included CIN 1 or low-grade squamous intraepithelial lesion (LSIL). High-grade histology included CIN 2 (stratification by p16 staining was not used given that it was not consistently performed throughout the study period), CIN 3, carcinoma in-situ, adenocarcinoma in-situ, or high-grade squamous intraepithelial lesion (HSIL).^20,21^ Abnormal cytology or HPV test results for which diagnostic testing or follow-up was recommended by the 2013 ASCCP cervical cancer screening management guidelines^22^ but for which there were no subsequent records were coded as unknown dysplasia and omitted from data analysis. For individuals with more than 1 cervical cytology test during the study period, data associated with the most significant diagnostic finding were used.

The primary end point was the PPV of cervical cytology for high-grade histology or cancer (CIN 2 or more severe diagnosis), ie, the proportion of participants who screened positive by abnormal cytology and actually had CIN 2 or a more severe diagnosis. Secondary objectives were PPV of cervical cytology for individuals completely vs incompletely vaccinated per ACIP guidelines and by age at vaccination initiation, dichotomized as younger than 21 years vs 21 years or older. The age cut point was chosen based on study results showing no significant risk reduction for CIN 2 or more severe diagnosis when vaccination was initiated at age 21 years or older.^4^

### Statistical Analysis

Automated data query identified 51 339 study participants. Initial data review excluded 335 participants who had no cervical cytology results documented. An additional 4016 participants were excluded because of missing or illogical dates for vaccination and/or screening tests, abnormal screening documented before or on the same day as vaccination, or a diagnosis of noncervical lower genital tract dysplasia (Figure).

**Figure.**
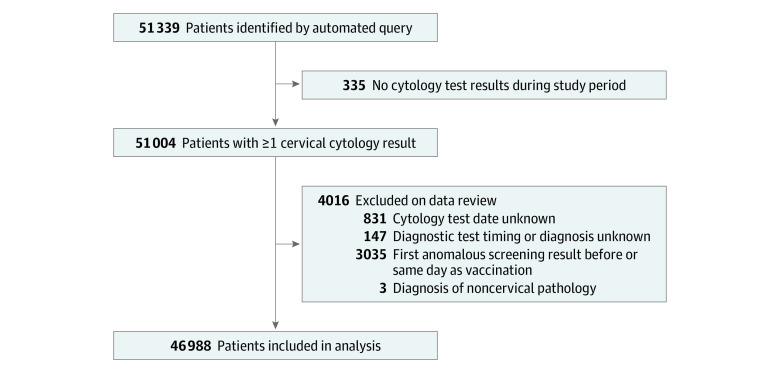
Study Flow Diagram

Descriptive statistics were used to describe patient demographic and clinical characteristics. The PPVs were compared by HPV vaccination status using logistic regression models adjusting for available confounding variables determined a priori, ie, age, race, and smoking status. Additional subgroup analyses among the vaccinated cohort were performed, focusing on comparisons by complete vs incomplete vaccination and dichotomized age at vaccination initiation. Point estimates and associated 95% CIs are presented. Data were analyzed using SAS version 9.4 (SAS Institute). *P* < .05 was considered statistically significant, and all tests were 2-tailed.

## Results

### Study Population

The final study population comprised 46 988 individual patient records (Figure). Mean (SD) age at the time of data abstraction was 28.7 (4.5) years. Most participants were White (35 446 [75.4%]) and non-Hispanic (43 399 [97.2%]); 3058 (6.5%) were Asian, and 4159 [9.6%] were Black. Less than 30% were current or former smokers (current: 5632 [12.0%]; former: 7653 [16.3%]) (Table 1).

**Table 1.** Participant Characteristics

Characteristic	Participants, No. (%) (N = 46 988)
Age at cytology, mean (SD), y	28.7 (4.5)
Race	
Asian	3058 (6.5)
Black or African American	4159 (8.9)
White	35 446 (75.4)
Other^a^	695 (1.5)
Missing	3630 (7.7)
Ethnicity	
Hispanic or Latina	1249 (2.7)
Not Hispanic or Latina	43 399 (97.2)
Missing	2340 (4.9)
Smoking status	
Smoker	
Current	5632 (12.0)
Former	7653 (16.3)
Never	33 222 (70.7)
Passive smoke exposure	400 (0.9)
Unknown	79 (0.2)

^a^ Other includes American Indian or Alaskan Native, Native Hawaiian or other Pacific Islander, other race not included in the given categories, and more than 1 race.

In the study population, 15 440 (33.0%) had received at least 1 dose of the HPV vaccine, and 11 644 (24.9%) were completely vaccinated. HPV vaccination coverage varied by race, with 562 Asian participants (18.4%), 1021 Black participants (24.5%), and 12 696 White participants (35.8%) vaccinated (*P* < .001) (eTable 1 in the Supplement). Participants who had completed the vaccine series were younger (mean [SD] age, 25.7 [3.8] years) compared with those who were incompletely vaccinated (27.1 [3.9] years) or unvaccinated (30.0 [4.2] years), suggesting increasing vaccination coverage over time. Among those who were vaccinated, 10 437 (67.6%) initiated vaccination at younger than 21 years of age, and 5003 (32.4%) initiated vaccination at 21 years or older.

### Cervical Cancer Screening Results

During the study period, 4289 participants (9.1%) had at least 1 abnormal cervical cytology result, with significant differences in prevalence of abnormal results by age, race, and smoking status (eTable 2 in the Supplement). When adjusted for these factors, complete vaccination was associated with higher odds of abnormal cervical cytology compared with no vaccination (OR, 1.08; 95% CI, 1.00-1.17) (Table 2). HPV test results were not analyzed given that neither cotesting nor HPV primary testing were recommended during the study period for individuals aged 21 to 29 years.

**Table 2.** Cervical Cytology Results by HPV Vaccination Status Among 46 988 Participants

HPV vaccination status	Participants, No. (%)		*P* value	OR (95% CI)	
	Abnormal^a^	Normal		Unadjusted^b^	Adjusted^b^^,^^c^
Unvaccinated	2785 (8.8)	28 709 (91.2)	<.001	1 [Reference]	1 [Reference]
Incompletely^d^	330 (8.7)	3484 (91.4)		0.98 (0.87-1.10)	0.89 (0.78-1.01)
Completely	1174 (10.1)	10 506 (90.0)		1.15 (1.07-1.24)	1.08 (1.00-1.17)

Abbreviations: HPV, human papillomavirus; OR, odds ratio.

^a^ Abnormal included any abnormal cytology result during the study period.

^b^ Compared with unvaccinated individuals.

^c^ Adjusted for age, race, and smoking status.

^d^ Incompletely vaccinated defined as receiving at least 1 dose of HPV vaccine but not receiving at least 2 doses if younger than 15 years or 3 doses if 15 years or older at time of vaccination initiation.

Prevalence of CIN 2 or more severe diagnosis among all study participants was 1.8% (855 participants). Prevalence among unvaccinated participants was 1.9% (594 participants) and 1.6% (187 participants) among those who were completely vaccinated. Initiating HPV vaccination when younger than 21 years was associated with decreased prevalence of CIN 2 or more severe diagnosis compared with vaccination initiation at age 21 years or older (1.4% [109 participants] vs 3.0% [78 participants]; *P* < .001).

Documented screening and diagnostic results were compared by the automated data query compared to NLP supplemented by manual chart review. NLP changed 609 histologic diagnoses (1.3%), with 327 (53.7%) upgraded and 282 (46.3%) downgraded. In a data set of nearly 50 000 patients, these changes did not significantly affect study conclusions.

### PPV of Cervical Cytology for CIN 2 or More Severe Diagnosis

Among the entire study population, the PPV of cervical cytology for CIN 2 or more severe diagnosis was 19.9% (95% CI, 19.3%-20.6%) (Table 3). PPV was significantly lower in the vaccinated group (17.4%; 95% CI, 16.4%-18.4%) compared with the unvaccinated group (21.3%; 95% CI, 20.4%-22.3%; *P* = .002). This difference seemed to be driven by the completely vaccinated group, where the PPV was 15.9% (95% CI, 14.9%-17.0%) compared with 22.4% (95% CI, 20.0%-25.0%; *P* = .003) in the incompletely vaccinated group. Age at vaccination initiation was associated with the greatest difference in PPV, with PPV of 11.9% (95% CI, 10.9%-12.9%) in the group initiating vaccination when younger than 21 years compared with 30.7%; (95% CI, 27.3%-34.4%; *P* < .001) when vaccination initiation was at 21 years of age or older. Additionally, the PPV among current (24.3%; 95% CI, 21.5%-27.3%) or former (27.4%; 95% CI, 24.7%-30.2%) vs never (16.8%; 95% CI, 15.7%-18.0%) smokers was higher in both the vaccinated (*P* = .004) and unvaccinated (*P* < .001) groups (eTable 3 in the Supplement).

**Table 3.** Cervical Histology and PPV Among Participants With Abnormal Cervical Cytology by Vaccination Status^a^

Result	Participants, No. (%)							
	All (n = 4289)	Vaccinated (n = 1504)	Unvaccinated (n = 2785)		Among vaccinated			
					Completely (n = 1174)	Incompletely (n = 330)^b^	Age at vaccine initiation, y	
							<21 (n = 920)	≥21 (n = 254)
Cancer	31 (0.7)	3 (0.2)		28 (1.0)	2 (0.2)	1 (0.3)	2 (0.2)	0
Histologic finding								
HSIL^c^	824 (19.2)	258 (17.2)		566 (20.3)	185 (15.8)	73 (22.1)	107 (11.6)	78 (30.7)
LSIL^d^	839 (19.6)	363 (24.1)		476 (17.1)	288 (24.5)	75 (22.7)	235 (25.5)	53 (20.9)
Negative	2595 (60.5)	880 (58.5)		1715 (61.6)	699 (59.5)	181 (54.8)	576 (62.6)	123 (48.4)
PPV, % (95% CI)^e^	19.9 (19.3-20.6)	17.4 (16.4-18.4)		21.3 (20.4-22.3)	15.9 (14.9-17.0)	22.4 (20.0-25.0)	11.9 (10.9-12.9)	30.7 (27.3-4.4)

Abbreviations: HSIL, high-grade squamous intraepithelial lesion; LSIL, low-grade intraepithelial lesion; PPV, positive predictive value.

^a^ Abnormal included any abnormal cervical cytology result during the study period.

^b^ Incompletely vaccinated defined as receiving at least 1 dose of HPV vaccine but not receiving at least 2 doses if younger than 15 years or 3 doses if 15 years or older at time of vaccination initiation.

^c^ Histologic HSIL included cervical intraepithelial neoplasia 2 or 3, carcinoma in-situ, or adenocarcinoma in-situ. Cervical intraepithelial neoplasia 2 was not stratified by p16 status because of inconsistent testing during the study period.

^d^ Histologic LSIL included only cervical intraepithelial neoplasia 1.

^e^ PPV of abnormal cytology to predict cervical intraepithelial neoplasia 2 or more severe diagnosis.

## Discussion

The results of our study show significantly lower PPV of cervical cytology for CIN 2 or more severe diagnosis among individuals vaccinated against HPV, even with HPV vaccination coverage of only 33%. Our results also suggest vaccinated should be defined as those who have completed vaccination per ACIP guidelines if screening recommendations are stratified by vaccination status. Lastly, our data suggest decreased screening intensity may only be appropriate for individuals initiating HPV vaccination when younger than 21 years.

Data from clinical trials have shown reduced geometric mean antibody titers of single vs multiple doses of the HPV vaccine, but determination of noninferiority was inconclusive; furthermore, it remains unknown at what titer adequate clinical efficacy is achieved.^23^ Observational studies have shown less of a difference in efficacy by number of doses received when vaccination was initiated prior to age 14 years.^23,24^ Data from our study suggest cervical cytology PPV is not significantly different between those who have been inadequately vaccinated and those who are unvaccinated. Thus, modifications to the cervical cancer screening guidelines for vaccinated individuals should be restricted to those who have completed the vaccine series per ACIP guidelines until we have additional data defining an age at vaccination initiation which may result in adequate clinical efficacy after a single dose.

The modest increase in odds of abnormal cytology associated with complete vaccination in our study is intriguing. Patients with documented abnormal screening results prior to or on the day of HPV vaccination were omitted from the study population, decreasing the risk of clinically significant HPV infection prior to vaccination. The adjusted analyses controlled for age at vaccination initiation, which is often used as a proxy for HPV exposure, but study methods did not allow for reliable collection of data on sexual debut, thus we cannot entirely discount the possibility that sexually active individuals were more motivated to complete the HPV vaccine series. We also acknowledge the possibility that vaccination may be associated with increased screening frequency and thus increased detection of transient abnormal cytology.^25,26^ Our findings are similar to results from a register-based cohort study from Denmark that showed no decrease in atypical squamous cells of undetermined significance–positive cytology in a vaccinated cohort compared with an unvaccinated cohort despite a decrease in HSIL cytology (and its inherent risk of CIN 2 or more severe diagnosis) in the vaccinated cohort.^3^ Likewise, a retrospective cohort study from Kaiser Permanente showed decreased accuracy of cervical cytology to predict current or 3-year risk of CIN 2 or more severe diagnosis.^27^ This means there is a significantly higher proportion of a false-positive screening results after vaccination. While the primary goal of cervical cancer screening is to detect clinically significant lesions and ultimately prevent cancer, the negative impact of false-positive results cannot be ignored. For individuals with an abnormal screening test, 50% to 80% will experience pain, bleeding, and/or discharge associated with diagnostic procedures or treatment.^28^ Studies have shown anxiety associated with an abnormal screening test result can last up to 24 months, even after a subsequent normal diagnostic test result.^29^ Lastly, false-positive screening tests add cost to the health care system with minimal additional health benefit.^30^ To date, HPV primary or cotesting has not been recommended in the youngest screening age group because of a high infection prevalence with subsequent clearance. However, as vaccination against HPV increases and the prevalence of HPV infection decreases, incorporation of HPV testing in this age group may need to be reconsidered, with focus on its negative predictive value (ie, identification of individuals at the lowest risk of CIN 2 or a more severe diagnosis) to identify a group requiring the least intensive screening strategy.

While the optimal age of HPV vaccination is 9 to 12 years, the HPV vaccine has been approved by the US Food and Drug Administration for female individuals as old as 26 years since 2006 and updated to age 45 years in 2018. However, clinical efficacy of the vaccine decreases as the age at vaccination initiation increases. This is demonstrated by the relatively high prevalence of CIN 2 and more severe diagnoses in the group vaccinated at age 21 years or older in our study, which likely minimizes the change in CIN 2 or more severe diagnosis prevalence among the larger vaccinated group. A case-control study showed the vaccine is most effective if initiated at 17 years or younger (CIN 2 or more severe diagnosis: rate ratio [RR], 0.45; 95% CI, 0.27-0.76), with no significant decrease in risk of CIN 2 or more severe diagnosis if vaccination was initiated at 21 years or older (RR, 0.92; 95% CI, 0.73-1.17).^4^ The importance of early vaccination to decrease cervical cancer incidence is further supported by population-based studies. A study from the United Kingdom showed vaccination initiation at age 12 to 13 years was associated with an 87% lower risk of cancer (adjusted incidence RR [aIRR], 0.13; 95% CI, 0.06-0.28) compared with a 34% risk reduction (aIRR, 0.66; 95% CI, 0.59-0.75) among those initiating vaccination at age 16 to 18 years.^9^ In a Swedish study, cervical cancer incidence among individuals vaccinated prior to 17 years of age was significantly less (aIRR, 0.12; 95% CI, 0.00-0.34) than for those vaccinated at age 17 to 30 years (aIRR, 0.47; 95% CI, 0.27-0.45).^5^ Concordant with these data, the findings of our study suggest that future guidelines for less intensive cervical cancer screening may only be indicated for individuals who have initiated HPV vaccination prior to age 21 years given that the PPV for cervical cytology is similar among those initiating vaccination at 21 years of age or older and those who are unvaccinated.

The HPV vaccination coverage reported in this study is low. Current HPV vaccination coverage in Minnesota is above the national average, with 81.9% of female individuals receiving at least 1 dose of the vaccine by age 17 years and 73.2% completing the HPV vaccine series.^13^ The lower vaccination coverage in this study is representative of the older population studied (ie, age 21-35 years at the time of data abstraction) and lower vaccination coverage in the early years of vaccine availability. Thus, our estimated decrease in the PPV of cervical cancer screening tests is likely conservative and is anticipated to further decrease as a larger proportion of vaccinated individuals age into screening. However, it is notable that in 2020 there were still states where vaccination coverage among female residents was less than 50%.^13^

### Strengths and Limitations

Strengths of our study include the large number of patient records with access to state immunization records. Our data review was thorough, comprising automated data query, NLP, and manual medical record review. This data abstraction process demonstrated that for a sufficiently large data set, automated data query may be sufficient, as the additional diagnostic information gained from the NLP was not substantial enough to change the study results.

This study also has limitations. Despite comprehensive medical record review, misclassification of data is still possible. Additional study limitations include those inherent to any retrospective study. Our data analyses were limited to results present in the system, and it is possible that screening frequency may differ by vaccination status. Among individuals with abnormal cervical cytology, 147 (0.3%) did not have a documented diagnostic evaluation and were ultimately omitted from the data analysis. Our data were limited to patients seen within a single health system with a predominately White, non-Hispanic population, and it is unclear whether these findings are generalizable to other populations. A majority of participants in our study received the quadrivalent HPV vaccine, and protection against CIN 2 and more severe diagnoses is anticipated to be even greater with increasing coverage with the nonavalent vaccine. The results of our study do not consider prevaccination HPV exposure or the vaccination status of the patients’ sexual partners, which may provide additional protection through herd immunity. Additionally, we are unable to comment on the predictive value of HPV testing because of the lack of cotesting or HPV primary testing in a large proportion of the study population.

## Conclusions

The results of our study show that despite only modest HPV vaccination coverage, PPV of cervical cytology for CIN 2 and more severe diagnoses was significantly lower among individuals who completed HPV vaccination, particularly when vaccination was initiated at younger than 21 years. While these results need to be confirmed in additional studies conducted among diverse populations in the United States, our results do suggest that delayed screening initiation and/or increased interval between screening tests as proposed by the modeling study by Kim et al^12^ should be restricted to individuals who initiated HPV vaccination prior to age 21 years and who have completed the recommended vaccine series.

## Notes

Supplement. **eTable 1.** HPV Vaccination Status by Patient Characteristics **eTable 2.** Cervical Cytology Results by Patient Characteristics **eTable 3.** Cervical Histology and Positive Predictive Value of Abnormal Cytology by Smoking Status
