# Supplementary material for: Test Performance of Cervical Cytology Among Adults With vs Without Human Papillomavirus Vaccination
Source: JAMA Netw Open. 2022 May 25;5(5):e2214020. doi: 10.1001/jamanetworkopen.2022.14020 (PMC9133945; doi:10.1001/jamanetworkopen.2022.14020)
Supplement: Supplement. — eTable 1. HPV Vaccination Status by Patient Characteristics eTable 2. Cervical Cytology Results by Patient Characteristics eTable 3. Cervical Histology and Positive Predictive Value of Abnormal Cytology by Smoking Status [file jamanetwopen-e2214020-s001.pdf]

Supplemental Online Content

Teoh D, Nam G, Aase DA, et al. Test performance of cervical cytology among adults with vs without human papillomavirus vaccination. *JAMA Netw Open.* 2022;5(5):e2214020. doi:10.1001/jamanetworkopen.2022.14020

eTable 1. HPV Vaccination Status by Patient Characteristics

eTable 2. Cervical Cytology Results by Patient Characteristics

eTable 3. Cervical Histology and Positive Predictive Value of Abnormal Cytology by Smoking Status

This supplemental material has been provided by the authors to give readers additional information about their work.

© 2022 Teoh D et al. *JAMA Network Open.*

eTable 1. HPV Vaccination Status by Patient Characteristics (N=46,988)				
	**Unvaccinated n=31,548 (67.1%)**	**Incompletely[a] vaccinated n=3,796 (8.1%)**	**Completely vaccinated n=11,644 (24.8%)**	
	**No. (%)**	**No. (%)**	**No. (%)**	**p-value**
**Age at cytology, Mean (SD)**	30.0 (4.2)	27.1 (3.9)	25.7 (3.8)	<0.0001
**Race**				<0.0001
Asian	2496 (7.9)	169 (4.4)	393 (3.4)	
Black or African American	3138 (9.9)	337 (8.9)	684 (5.9)	
White	22750 (72.1)	2949 (77.7)	9747 (83.7)	
Other[b]	464 (1.5)	64 (1.7)	167 (1.4)	
Missing	*2700 (8.6)*	*277 (7.3)*	*653 (5.6)*	
**Ethnicity**				<0.0001
Hispanic or Latina	913 (2.9)	115 (3.0)	221 (1.9)	
Not Hispanic, not Latina	28778 (91.2)	3539 (93.2)	11082 (95.2)	
Missing	*1857 (5.9)*	*142 (3.8)*	*341 (2.9)*	
**Smoking Status**				<0.0001
Current smoker	3613 (11.5)	625 (16.4)	1394 (11.9)	
Former smoker	5310 (16.9)	739 (19.4)	1604 (13.7)	
Never smoker	22327 (70.9)	2398 (62.9)	8497 (72.8)	
Passive smoke exposure	174 (0.6)	48 (1.3)	180 (1.5)	
Unknown	*70 (0.2)*	*4 (0.1)*	*5 (0.0)*	

HPV, human papillomavirus; SD, standard deviation

[a]Incompletely vaccinated defined as receiving at least 1 dose of HPV vaccine, but did not receive at least 2 doses if <15 years or 3 doses if 15 years or older at time of vaccination initiation.

[b]Other includes American Indian or Alaskan Native, Native Hawaiian or other Pacific Islander, other race not included in the given categories, or >1 race.

© 2022 Teoh D et al. *JAMA Network Open.*

eTable 2. Cervical Cytology Results by Patient Characteristics (N=46,988)

	Abnormal[a] n=4,289 (9.1%)	Normal n=42,699 (90.9%)	
	**No. (%)**	**No. (%)**	**p-value**
**Age at Cytology, Mean (SD)**	28.2 (4.5)	28.7 (4.5)	<0.0001
**Race**			<0.0001
Asian	204 (4.8)	2854 (6.7)	
Black or African American	428 (10.0)	3731 (8.7)	
White	3268 (76.2)	32178 (75.4)	
Other[b]	75 (1.7)	620 (1.4)	
Missing	*314 (7.3)*	*3316 (7.8)*	
**Ethnicity**			0.81
Hispanic or Latina	112 (2.6)	1137 (2.7)	
Not Hispanic, not Latina	3976 (92.7)	39423 (92.3)	
Missing	*201 (4.7)*	*2139 (5.0)*	
**Smoking Status**			<0.0001
Current smoker	713 (16.6)	4919 (11.5)	
Former smoker	822 (19.2)	6831 (16.0)	
Never smoker	2712 (63.2)	30510 (71.5)	
Passive smoke exposure	36 (0.8)	366 (0.9)	
Unknown	*6 (0.1)*	*73 (0.2)*	

SD, standard deviation

[a]Abnormal included any abnormal cytology result during the study period.

[b]Other includes American Indian or Alaskan Native, Native Hawaiian or other Pacific Islander, other race not included in the given categories, or >1 race.

© 2022 Teoh D et al. *JAMA Network Open.*

eTable 3. Cervical Histology and Positive Predictive Value of Abnormal Cytology by Smoking Status

				Among Unvaccinated			Among Vaccinated[a]		
**Smoking status**	**Current**	**Former**	**Never**	**Current**	**Former**	**Never**	**Current**	**Former**	**Never**
**Abnormal cytology[b]**	713	822	2712	478	567	1721	235	255	991
	**No. (%)**	**No. (%)**	**No. (%)**	**No. (%)**	**No. (%)**	**No. (%)**	**No. (%)**	**No. (%)**	**No. (%)**
**Cancer**	6 (0.8)	7 (0.9)	18 (0.7)	6 (1.3)	7 (1.2)	15 (0.9)	0 (0.0)	0 (0.0)	3 (0.3)
**Histologic HSIL[c]**	167 (23.4)	218 (26.5)	438 (16.2)	121 (25.3)	157 (27.7)	288 (16.7)	46 (19.6)	61 (23.9)	150 (15.1)
**Histologic LSIL[d]**	131 (18.4)	135 (16.4)	563 (20.8)	75 (15.7)	90 (15.9)	308 (17.9)	56 (23.8)	45 (17.6)	255 (25.7)
**Negative histology**	409 (57.4)	462 (56.2)	1693 (62.4)	276 (57.7)	313 (55.2)	1110 (64.5)	133 (56.6)	149 (58.4)	583 (58.8)
**PPV % (95% CI)**	24.3 (21.5-27.3)	27.4 (24.7-30.2)	16.8 (15.7-18.0)	26.6 (23.1-30.3)	28.9 (25.7-32.4)	17.6 (16.2-19.1)	19.6 (15.3-24.7)	23.9 (19.5-29.0)	15.4 (13.7-17.3)

CI, confidence interval; HSIL, high grade squamous intraepithelial lesion; LSIL, low grade intraepithelial lesion; PPV, positive predictive value

[a]Incompletely vaccinated defined as receiving at least 1 dose of HPV vaccine, but did not receive at least 2 doses if <15 years or 3 doses if 15 years or older at time of vaccination initiation.

[b]Abnormal included any abnormal cervical cytology result during the study period.

[c]Histologic HSIL included cervical intraepithelial neoplasia (CIN) 2, CIN 3, carcinoma in situ, or adenocarcinoma in situ. CIN 2 not stratified by p16 status due to inconsistent testing during the study period.

[d]Histologic LSIL included only CIN1.

[e]PPV of abnormal cytology to predict CIN 2+.

© 2022 Teoh D et al. *JAMA Network Open.*
